# Impact of concomitant idiopathic pulmonary fibrosis on prognosis in lung cancer patients: A meta-analysis

**DOI:** 10.1371/journal.pone.0259784

**Published:** 2021-11-12

**Authors:** Haoyu Wang, Ruiyuan Yang, Jing Jin, Zhoufeng Wang, Weimin Li

**Affiliations:** Department of Respiratory and Critical Care Medicine, West China Hospital, West China Medical School, Sichuan University, Chengdu, Sichuan, China; Shuguang Hospital, CHINA

## Abstract

**Background:**

Current studies showed that idiopathic pulmonary fibrosis (IPF) may lead to a poor prognosis of lung cancer. We conducted a meta-analysis to explore the impact of concomitant IPF in lung cancer and its prognostic value.

**Methods:**

We searched the databases of PubMed, Web of Science, Embase up to Feb 10^th^, 2021 for relevant researches and merged the hazard ratios (HRs) and 95% confidence intervals (CIs) to evaluate the association between concomitant IPF and overall survival (OS) in patients with lung cancer.

**Results:**

Twelve studies involving 58424 patients were included in our meta-analysis. The results indicated that concomitant IPF was correlated with poor prognosis of lung cancer patients (HR = 1.99, 95%CI, 1.59–2.51). The association remained consistent after subgroup analysis and meta-regression stratified by study region, sample size, tumor histology, and therapy. In addition, our results were robust even after sensitivity analysis.

**Conclusions:**

Concomitant IPF may be a prognostic factor of lung cancer, which can lead to poor survival. However, further studies were necessary for evidence in clinical application.

## Introduction

Novel cancer statistics revealed that lung cancer is still a common form of cancer and the leading cause of cancer death with a terribly high incident and mortality rate [1]. Despite the advances in diagnostic and therapeutic strategies, the prognosis of lung cancer patients remains extremely poor with a 5-year relative survival rate less than 20% [1, 2]. Idiopathic pulmonary fibrosis (IPF) is a form of chronic, progressive, fibrosing interstitial pneumonia of unknown etiology [3]. IPF is characterized by usual interstitial pneumonia (UIP) pattern in high-resolution computed tomography (HRCT) and histopathology [4]. Its incidence is 3–9 cases per 100000 per year for Europe and North America and is growing through time and age [5, 6]. A majority of IPF patients have an extremely poor prognosis whose 5-year survival rate is 20%-40% and median survival is 3–4 years though antifibrotic drugs like pirfenidone and nintedanib have already been applied to clinical practice [6–10]. Due to the high incidence and mortality rate, IPF is regarded as a tumor-like disease [11, 12].

IPF has many comorbidities and complications such as pulmonary hypertension (PH) [13, 14], obstructive sleep apnea syndrome (OSAS) [15, 16], emphysema [17], gastroesophageal reflux disease (GERD) [18, 19], coronary heart disease (CHD) [20, 21] and lung cancer is the most severe one of them [22, 23]. Previous studies confirmed that IPF might be a risk factor of lung cancer with a high prevalence and incidence rate [24–26]. In addition, lung cancer in IPF patients was more frequently in older male smokers and more likely to be squamous cell carcinoma (SQCC) [27]. Besides, current studies revealed the impact of idiopathic pulmonary fibrosis on the clinical outcome of lung cancer patients [28], but a comprehensive evaluation hasn’t been performed yet. Thus we searched a variety of publications and conducted a meta-analysis in order to evaluate the impact of concomitant IPF on lung cancer patients and help to improve the clinical strategies for these patients.

## Methods

### Protocol and registration

This meta-analysis is reported in accordance with the Preferred Reporting Items for Systematic Review and Meta-Analyses (PRISMA) statement [29] and was registered at International Prospective Register of Systematic Reviews (PROSPERO): number CRD42021235758 **(S1 File)**.

### Search strategy

We performed a systematic search in the following databases, Web of Science, Embase, and PubMed for cohort studies and case-control studies evaluating the association between coexistent idiopathic pulmonary fibrosis and the prognosis of lung cancer patients until Feb 10th, 2021, without any restrictions of language or publication status. The following keywords were used: “idiopathic pulmonary fibrosis”, “IPF”, “lung cancer”, “pulmonary tumor”, “lung carcinoma”, “prognosis”, “survival”. In addition, we also searched for potential articles from the reference lists of relevant articles manually. The search strategy for PubMed was presented in **S2 File**.

### Eligibility criteria

The following inclusion criteria were used: 1) all patients were pathologically diagnosed with lung cancer; 2) the outcomes included OS with a hazard ratio (HR) and corresponding 95% confidence interval (95% CI); 3) full-text papers published in English.

The exclusion criteria were as follows: 1) reviews, conference abstracts, cases reports, letters, or comments; 2) laboratory researches of clinical samples, animals, or cell lines; 3) insufficient data for estimating hazard ratios of concomitant IPF; 4) lack of control.

### Data extraction

Two researchers extracted the following data independently from the eligible studies, including family names of the first author, year of publication, study design, region, follow-up (months), sample size, therapy, and survival data. Any disagreement was resolved by discussion and consensus.

### Risk of bias assessment

The risk of bias of each enrolled study was assessed by applying the Newcastle-Ottawa quality assessment Scale (NOS) [30]. Studies labeled with 6 points or higher were considered high-quality studies. The quality assessment for each study was presented in S1 Table.

### Statistical analysis

Statistical analysis was conducted via R (version 4.0.3). HRs from multivariate analysis were used wherever available, and HRs from univariate models were substitutes if only univariate analysis was performed. Moreover, HRs and corresponding 95% CIs were estimated from Kaplan–Meier curves via the method by Tierney et al [31], if they weren’t provided directly. Pooled HRs and 95% CIs were combined with Mantel-Haenszel method according to a random effects model. Heterogeneity was assessed by forest plots, Chi^2^, I^2^, and Tau^2^ statistics. The significant heterogeneity was defined as p-value < 0.05 and I^2^ > 50%. Sensitivity analysis was conducted by excluding each study independently from our meta-analysis to find out the over-representation of every single study. Subgroup analysis and meta-regression were performed to investigate potential confounding factors of this meta-analysis. Publication bias was evaluated by Begg’s test, funnel plots, and Baujat plot [32], and a trim and fill method [33] was used to modify our meta-analysis when publication bias was obvious. P-value < 0.05 was considered as being statistically significant.

## Results

### Literature search and risk of bias assessment

**Fig 1:**
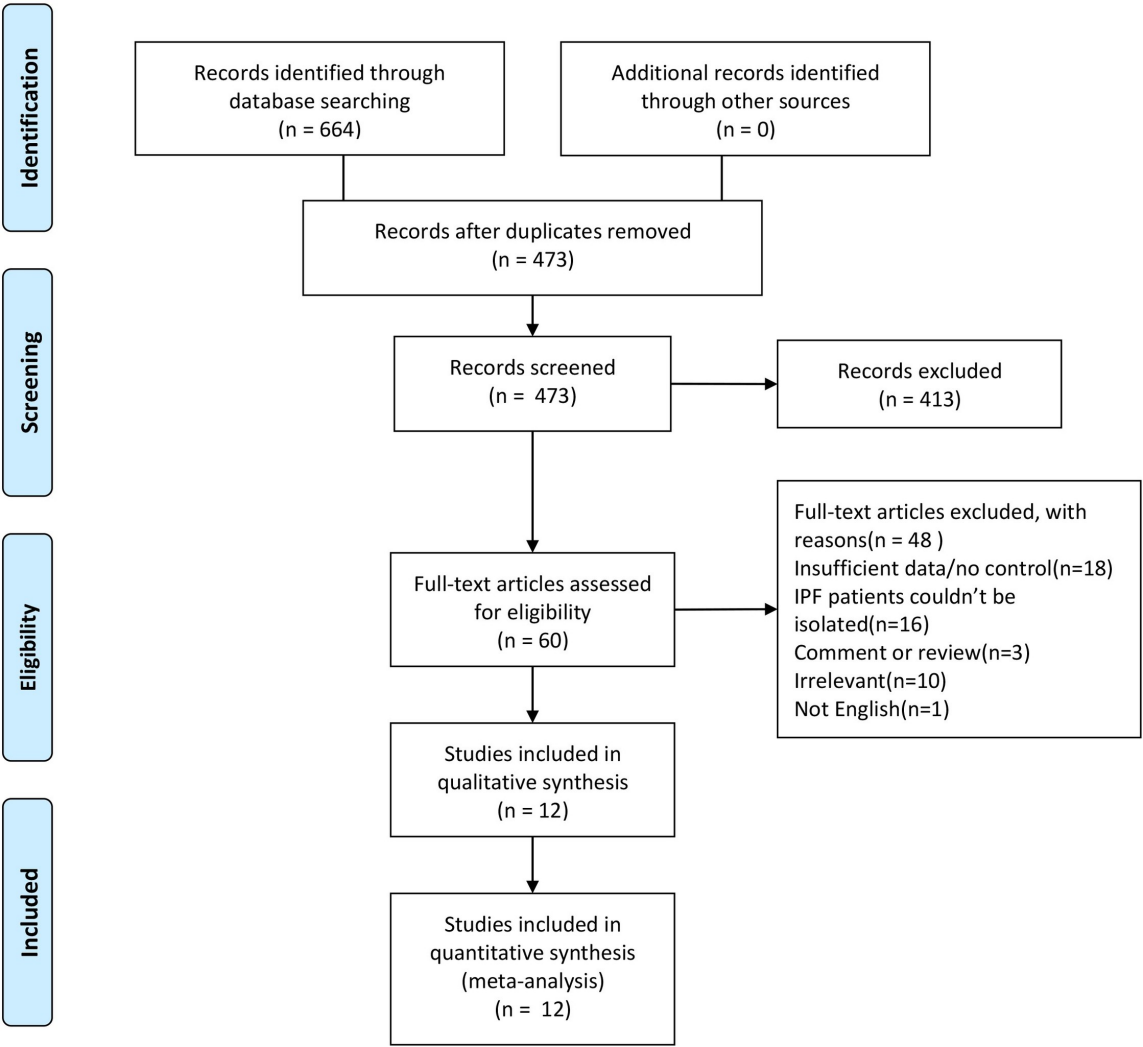
PRISMA flow diagram of the literature search in this meta-analysis.

The PRISMA flow diagram and checklist of this meta-analysis were presented in **Fig 1** and **S3 File**. A total of 664 separate publications were retrieved through our search strategy. We reduced these to a list of 473 articles after removing duplicates. We found 60 potentially eligible studies according to titles and abstracts and then screened full-text versions of them. Finally, twelve studies were included in this meta-analysis. The NOS scores varied from 7 to 9, which demonstrated a low risk of bias in these studies.

### Characteristics of included studies

The main characteristics of all 12 studies that met our inclusion and exclusion criteria were displayed in **Table 1** [34–45].

**Table 1:** Main characteristics of the studies included in this meta-analysis.

					Age		Gender (M/F)						
Author	Publication date	Study design	Study Region	Sample Size	IPF	Non-IPF	IPF	Non-IPF	TNM Stage	Tumor histology	Therapy	Follow-up(months)	NOS
Aubry	2002 Aug	RO	USA	556	72.1±9.7	69.5±8.6	21/3	345/187	I-IIIa	NSCLC	Surgery	0.2–73.2	7
Kawasaki	2002 Sep	RO	Asia	711	66 (54–80)	64 (22–89)	49/4	409/249	I-IV	NSCLC	Surgery	4–85	7
Watanabe	2008 Sep	RO	Asia	858	68±7	69±5	50/6	517/285	I-IV	NSCLC	Surgery	NA	7
Saito	2011 Nov	RO	Asia	350	70.4±6.54	64.5±10.9	4/24	176/146	Ia	NSCLC	Surgery	0–187.2	8
Goto	2014 Apr	RO	Asia	387	73.3±6.4	69.4±10.4	56/9	196/126	I-IV	NSCLC	Surgery	NA	8
Lee	2014 Aug 15	RO	Asia	99	67±8	66±7	31/2	62/4	I-IIIa	NSCLC	Surgery	0–95	8
Kanaji	2016 Jun 27	RO	Asia	199	70 (57–86)	66 (30–91)	34/0	95/70	IIIb-IV	NSCLC	Target therapy	NA	7
Kim	2019 Oct 4	RO	Asia	86	74.5 (72.0–79.0)^a^	78.5 (74.0–81.5)	22/0	34/30	I-II	NSCLC	Radiotherapy	1–92	7
Koyama	2019 Aug 23	RO	Asia	93	72±7.1	68±7.8	15/5	57/16	LD, ED	SCLC	Chemotherapy	NA	8
Brown	2019 Aug	RO	Asia	54453	76 (71–81)^a^	74 (69–80)	515/340	28106/25492	I-IV	NSCLC	NA	NA	8
Song	2020 Jun 29	RO	USA	288	69.7±7.4	69.0±7.6	86/10	169/23	I-III	NSCLC	Surgery	49.2^b^	9
Kanaji	2020 Oct 21	RO	Asia	344	73 (52–87)	68 (27–89)	71/4	234/35	LD, ED	SCLC	Chemotherapy	NA	7

RO: Retrospective; TNM: Tumor, node, metastasis; USA: United States of America; NSCLC: Non-small cell lung cancer; SCLC: Small cell lung cancer; LD: Limited disease; ED: Extensive disease; NA: Not available.

a: Reported as median (interquartile range, IQR). Other studies were reported as mean± standard deviation (SD) or median (range).

b: Reported as median. Other studies were reported as range.

Of all included studies, 10 were conducted in Asia [35–42, 44, 45] and the rest were in the United States of America (USA) [34, 43]. All 12 studies were retrospective, consist of 1 case-control study [39] and 11 cohort studies [34–38, 40–45]. The sample size ranged from 86 to 54453. Of all studies, 10 enrolled patients with NSCLC [34–38, 40, 41, 43, 45] while 2 enrolled SCLC patients [42, 44]. Patients of 7 studies received surgery [34–39, 45] while 4 studies received chemotherapy, radiotherapy, or target therapy [40–42, 44]. All studies defined OS as the time from diagnosis to the day of death or last follow-up.

### Meta-analysis result of OS

**Fig 2:**
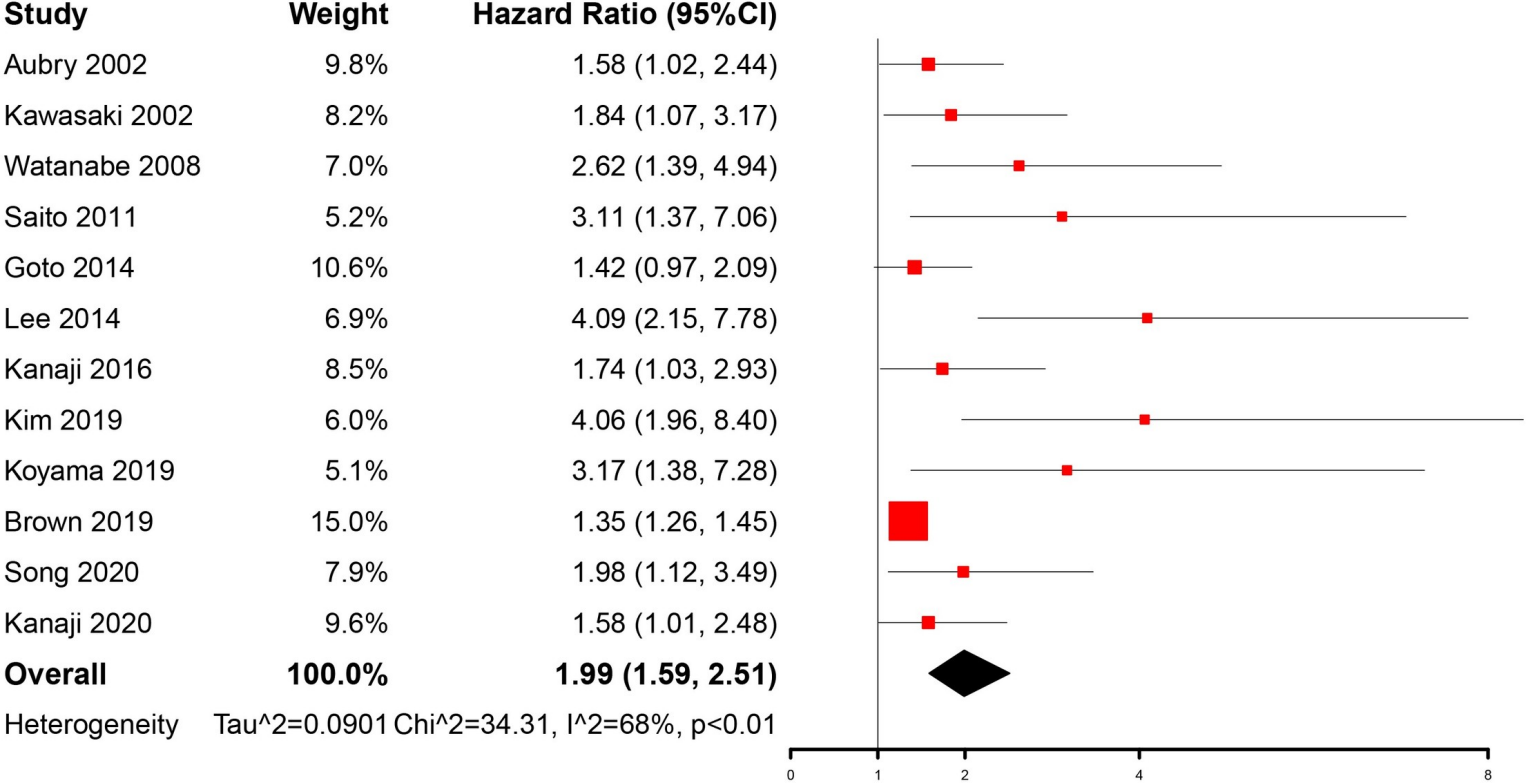
The forest plot of association between concomitant IPF and OS in lung cancer patients.

12 studies involving a total of 58424 patients (1361 with IPF and lung cancer and 57063 with lung cancer only) contributed to the primary analysis (**Fig 2**). The result indicated that concomitant IPF is associated with OS of lung cancer patients (HR = 1.99, 95%CI, 1.59–2.51). We found significant heterogeneity among studies (I^2^ = 68%, p <0.01).

### Subgroup analyses

**Fig 3:**
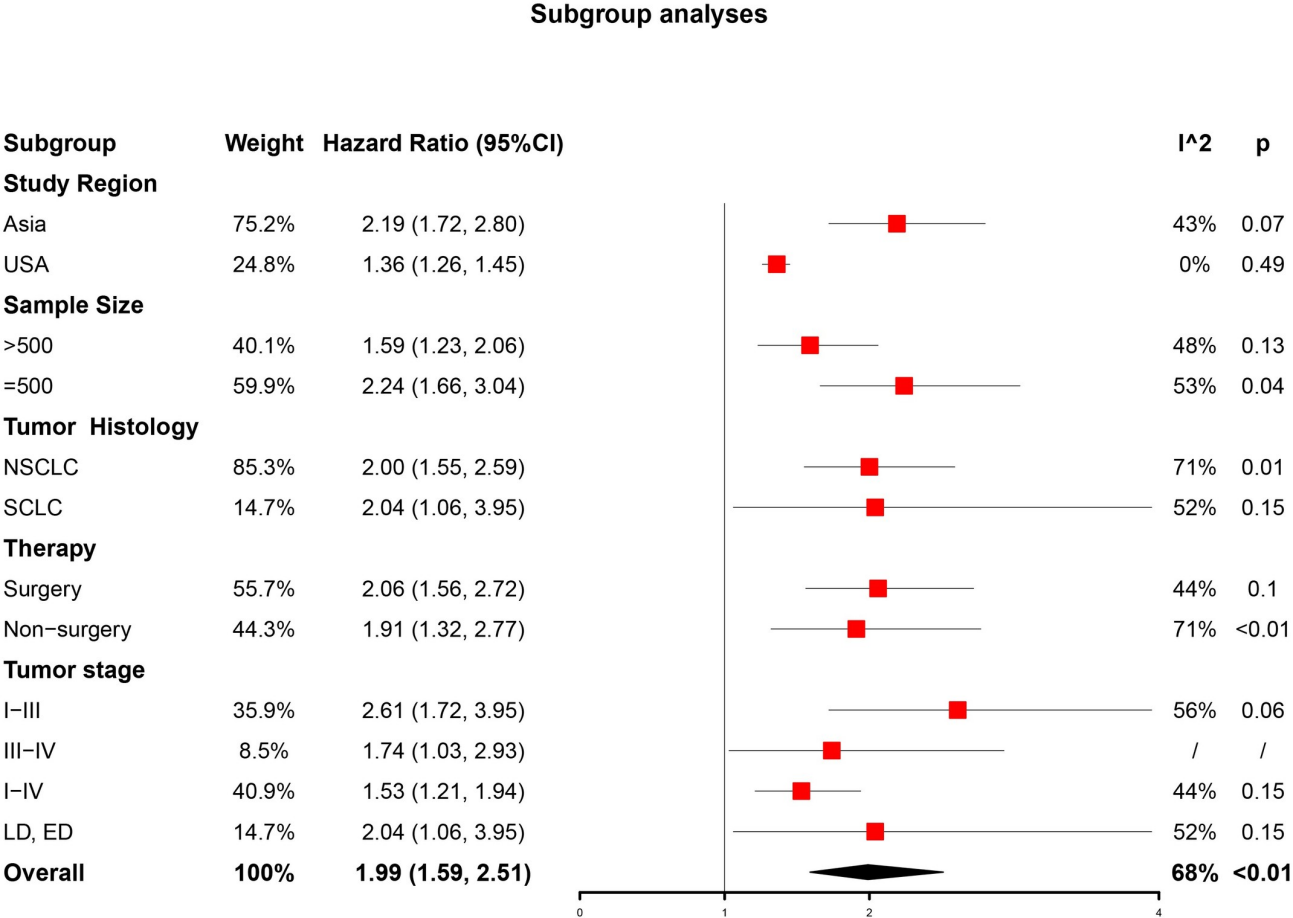
Subgroup analyses of association between concomitant IPF and OS in different population.

In order to detect the potential cause of heterogeneity, we performed subgroup analyses stratified by study region, sample size, tumor histology, and therapy (**Fig 3**). In the analysis of the study region, patients from Asia and USA showed both insignificant heterogeneities, and the results were (HR = 2.19, 95%CI, 1.72–2.80, I^2^ = 43%, p = 0.07) and (HR = 1.36, 95%CI, 1.26–1.45, I^2^ = 0%, p = 0.49), respectively. In the analysis of sample size, studies with sample size of more than 500 [34–36, 43] showed insignificant heterogeneity (HR = 1.59, 95%CI, 1.23–2.06, I^2^ = 48%, p = 0.13) whereas studies with a sample size of no more than 500 [37–42, 44, 45] still had significant heterogeneity (HR = 2.24, 95%CI, 1.66–3.04, I^2^ = 53%, p = 0.04). Similarly, patients receiving surgery had insignificant heterogeneity (HR = 2.06, 95%CI, 1.56–2.72, I^2^ = 44%, p = 0.10) while non-surgery patients were opposite (HR = 1.91, 95%CI, 1.32–2.77, I^2^ = 71%, p<0.01). For the tumor stage, the results for all subgroups were significant, but there weren’t any differences among all subgroups, and only the subgroup of Stage I-IV was with low heterogeneity after stratification (HR = 1.53, 95%CI, 1.21–1.94, I^2^ = 44%, p = 0.15).

### Meta-regression

Subsequently, we performed meta-regression to quantificationally analyze the potential source of heterogeneity (**Table 2**). In the univariate model, the study region appeared to be the main source of heterogeneity (p = 0.04, b = -0.42, 95%CI, -0.83- -0.01), however, when performing multivariate analysis, the significance disappeared (p = 0.3427, b = -0.4107, 95%CI, -1.25–0.44).

**Table 2:** Result of meta-regression.

Variable	Univariate				Multivariate			
	SE	b	95%CI	p	SE	b	95%CI	p
Study Region	0.21	-0.42	(-0.83, -0.01)	0.04	0.43	-0.41	(-1.26, 0.44)	0.34
Sample Size	0.22	-0.29	(-0.71, 0.14)	0.18	0.37	-0.07	(-0.79, 0.65)	0.85
Tumor Histology	0.34	0.01	(-0.66, 0.68)	0.98	0.42	-0.14	(-0.96,0.67)	0.73
Therapy	0.23	0.11	(-0.35, 0.56)	0.64	0.30	0.00	(-0.60, 0.59)	0.99

SE: Standard error; 95%CI: 95% confidence interval.

### Sensitivity analysis

**Fig 4:**
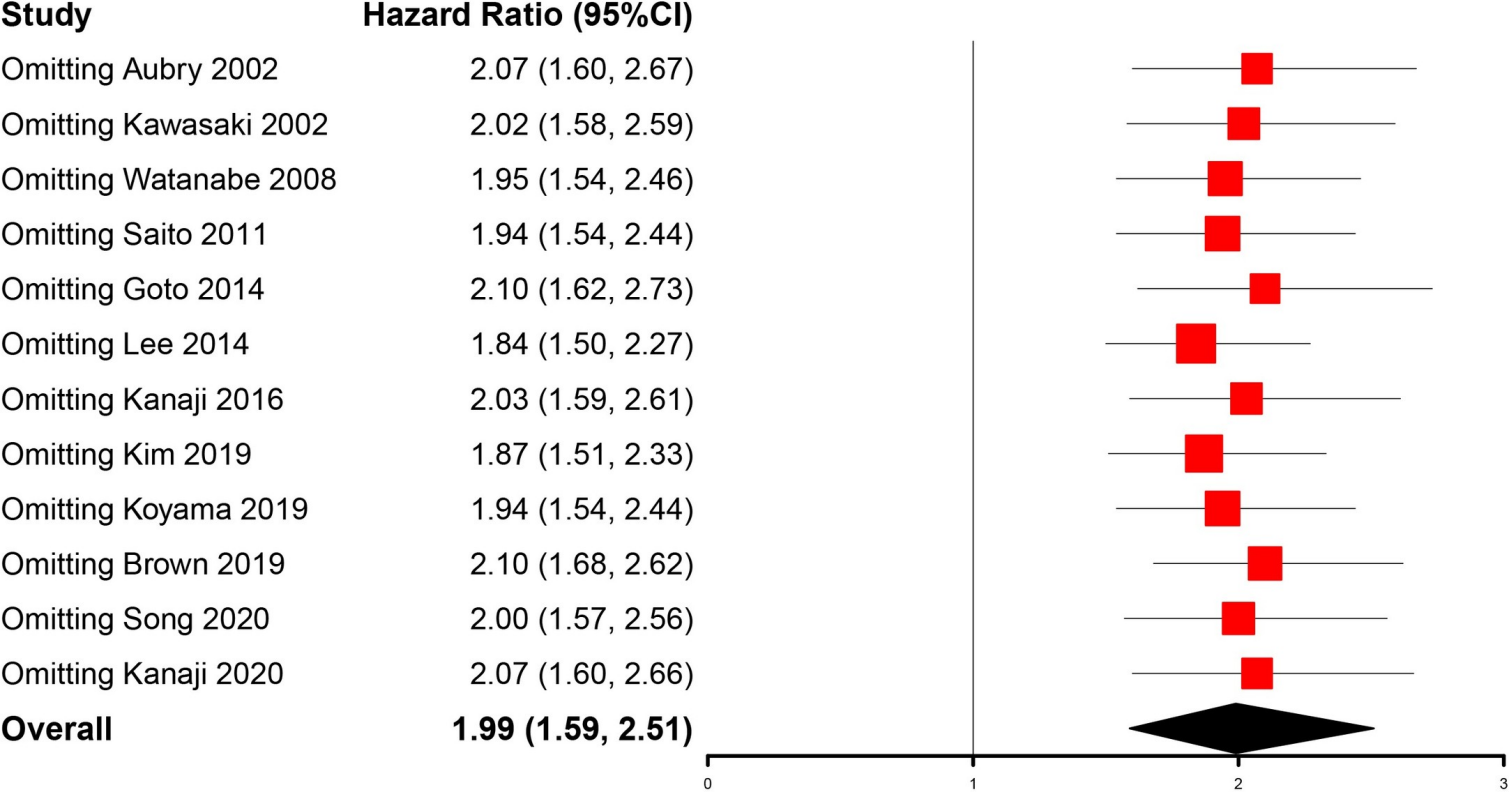
Sensitivity analysis by excluding each study from meta-analysis.

We then conducted a sensitivity analysis to evaluate the impact of every single study on the combined HRs by excluding each study independently from the meta-analysis. All HRs were in the 95%CI of our primary analysis, which demonstrated that our pooled HRs for OS were robust (**Fig 4**).

### Publication bias

**Fig 5:**
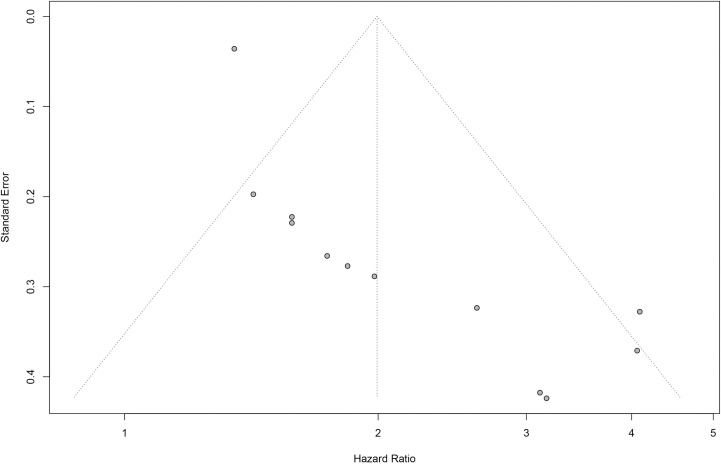
Funnel plot for detecting publication bias of studies included.

**Fig 6:**
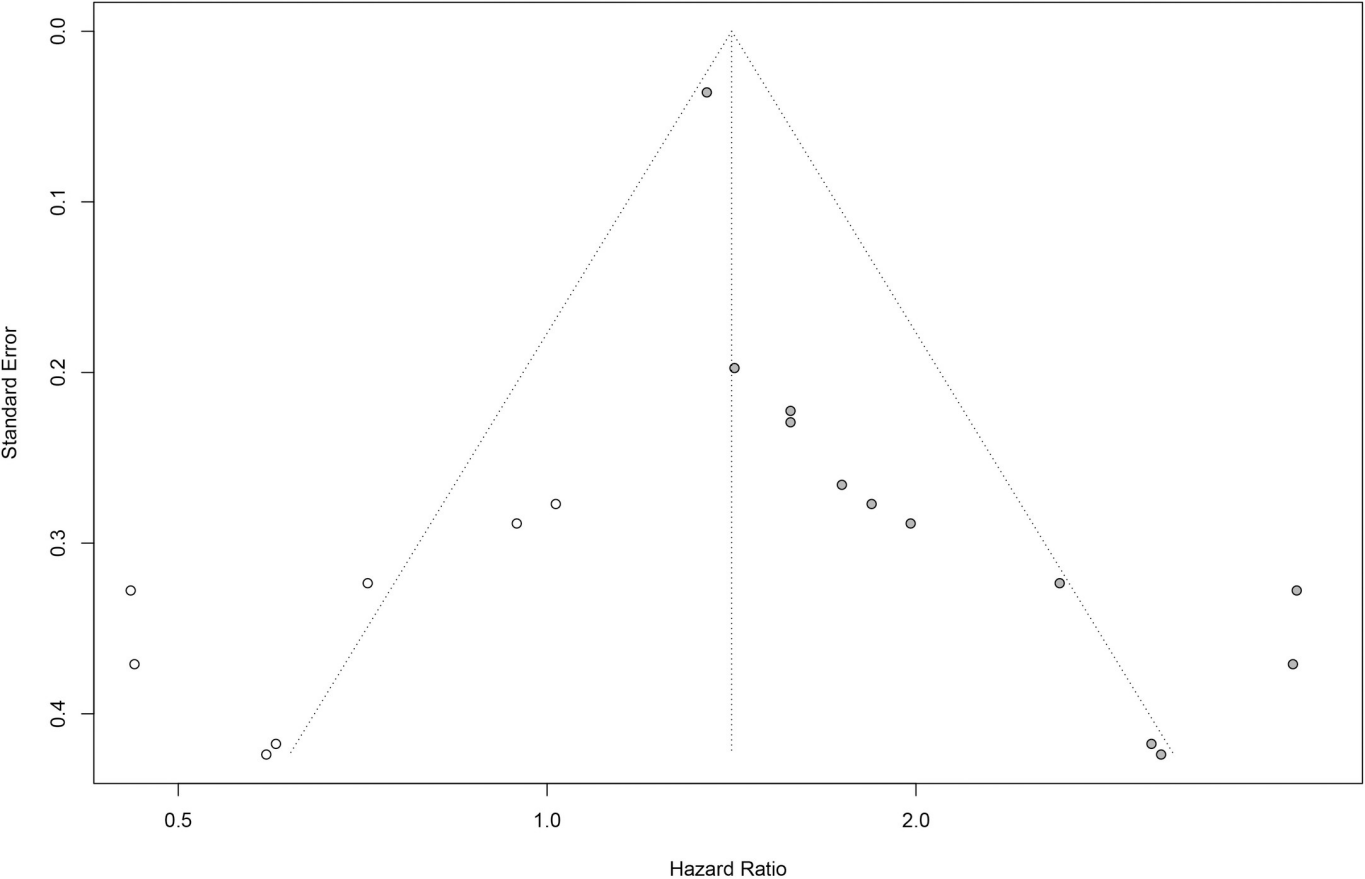
Funnel plot after a trim and fill analysis.

Finally, we conducted Begg’s test and drew funnel plots to assess the publication bias of all included studies (**Fig 5**). The p-value of Begg’s test was less than 0.01, which showed an indication of publication bias. So we drew a Baujat plot to detect the source of bias and heterogeneity (**S1 Fig**) and we subsequently conducted a meta-analysis after removing two studies with possibly high heterogeneity (**S2 Fig**). We then conducted a trim and fill analysis (**Fig 6**). The study of Lee contributed most to heterogeneity while Brown had the highest influence on the overall result. The results after a trim and fill analysis were still significant (HR = 1.41, 95%CI, 1.14–1.76).

## Discussion

Our current meta-analysis suggested that concomitant IPF may be associated with poor prognosis of patients with lung cancer (HR = 1.99, 95%CI, 1.59–2.51). However, the I2 and p-value indicated that there was high heterogeneity among the studies included (I^2^ = 68%, p <0.01). Then we attempted to find out the source of heterogeneity by conducting subgroup analyses and meta-regression. The subgroup analyses revealed that concomitant IPF could consistently be a prognostic factor of lung cancer patients when stratified by study region, sample size, tumor histology, and therapy. In addition, the results also showed that stratification according to study region, sample size, and therapy may reduce the heterogeneity, which suggested these factors might contribute to the heterogeneity of studies. However, the results of meta-regression demonstrated that the study region, sample size, and therapy may not be the main source of heterogeneity even though the study region displayed statistical significance in univariate analysis. Interestingly, when analyzing the impact of tumor histology, we found that both NSCLC and SCLC subgroups remained high heterogeneity and maybe it’s due to the subtypes of histology. Furthermore, our meta-analysis proved to be robust after sensitivity analysis. Although there was publication bias, which meant some studies with negative results may not be published, the result modified by a trim and fill method confirmed that concomitant IPF had identical prognostic value in patients with lung cancer.

Previous studies suggested that IPF and cancer are similar and multiple pathological processes were involved, for instance, genetic or epigenetic alterations and abnormal cell phenotypes like proliferation and apoptosis exist in both IPF and cancer [11]. For lung cancer, IPF has much consistent pathogenic mechanism though its etiology remains obscure. Current studies revealed that Tumor Protein P53 (TP53), Mesenchymal Epithelial Transition Proto-Oncogene (MET), B-Raf Proto-Oncogene (BRAF), and Kirsten Rat Sarcoma Viral Oncogene (KRAS), which were commonly altered genes of lung cancer, were mutant or upregulated in IPF patients [46, 47]. Moreover, common signaling pathways also play roles in both diseases, for example, deregulated PIK3/AKT pathway contributed to IPF and lung cancer by activating downstream molecules like Transforming Growth Factor Beta 1 (TGF-β_1_), which is known as a profibrotic mediator [48, 49]. In addition, the antifibrotic drug nintedanib appeared to benefit the overall survival of lung cancer patients [50], which indicates that there might be some common therapeutic targets of lung cancer and IPF. For the clinical aspect, patients with IPF tend to have worse lung function than those without IPF [3], characterized by restrictive ventilatory disorder and diffusion dysfunction. Therefore, lung cancer patients with concomitant IPF may have a higher risk for and dyspnea and acute exacerbation, which can lead to a worse outcome of survival. Moreover, the main treatment at present for IPF includes steroids, immunosuppressants, and antifibrotic medications [51], which can strongly suppress the immune system of patients, and thus lung cancer patients with concomitant IPF also gain a higher risk of infection or dysbiosis due to their treatment, which was proved to affect the prognosis of lung cancer [52, 53]. In general, concomitant IPF may promote development and progression through underlying mechanisms that were formerly mentioned.

Our study conclusion was partly similar to those of another study by JafariNezhad [27], however, they focused more on whether IPF is a risk factor of lung cancer and our meta-analysis was the first to evaluate the prognostic value of IPF in lung cancer patients. Likewise, studies included in our meta-analysis were strictly selected by our inclusion and exclusion criteria, although several studies had sufficient data they were excluded because IPF couldn’t be isolated from interstitial lung disease (ILDs) [54–60], which may increase the heterogeneity and bias.

However, the current research has some limitations, too. The most obvious one is that there was high heterogeneity and publication bias. The heterogeneity was considerably reduced after omitting studies of Lee and Kim without attenuating the pooled HRs, which suggested that these two studies may be the main source of heterogeneity although they had no significant bias after our reassessment (S2 Fig). Our results remained stable despite the trim and fill analysis for minimizing the publication. Secondly, all studies were retrospective ones and some studies didn’t provide HR and corresponding 95%CIs, which may contribute to high heterogeneity. Last, some potential confounders that can influence the prognosis of lung cancer patients such as the experience of acute exacerbation, the lung function, and the treatment for IPF vary among all included studies, whereas we couldn’t adjust them by either subgroup analysis or meta-regression due to the lack of data.

## Conclusion

Generally speaking, our meta-analysis demonstrated the prognostic value and adverse effect of concomitant IPF in lung cancer patients. Nevertheless, our findings must be applied carefully and discreetly and more prospective cohort studies are required.

## Supporting information

S1 Fig Baujat plot for detecting contribution of studies to the overall result and heterogeneity. (TIF)

S2 Fig The forest plot of association between concomitant IPF and OS after excluding high heterogeneity studies. (TIF)

S3 Fig Detailed forest plots of subgroup analyses. The subgroup analyses stratified by (a) study region, (b) sample size, (c) tumor histology, (d) therapy, and (e) tumor stage.
(TIF)

S1 Table Quality assessment for each individual study assessed. (DOCX)

S1 File The protocol registered in International Prospective Register of Systematic Reviews. (PDF)

S2 File Search strategy for meta-analysis of impact of concomitant idiopathic pulmonary fibrosis on prognosis in lung cancer patients (PubMed via NLM). (DOCX)

S3 File PRISMA checklist. (DOC)

S4 File The minimal data set necessary to replicate our findings. (XLSX)
