# Supplementary figures and images for: Impact of concomitant idiopathic pulmonary fibrosis on prognosis in lung cancer patients: A meta-analysis
Source: PLoS One. 2021 Nov 12;16(11):e0259784. doi: 10.1371/journal.pone.0259784 (PMC8589161; doi:10.1371/journal.pone.0259784)

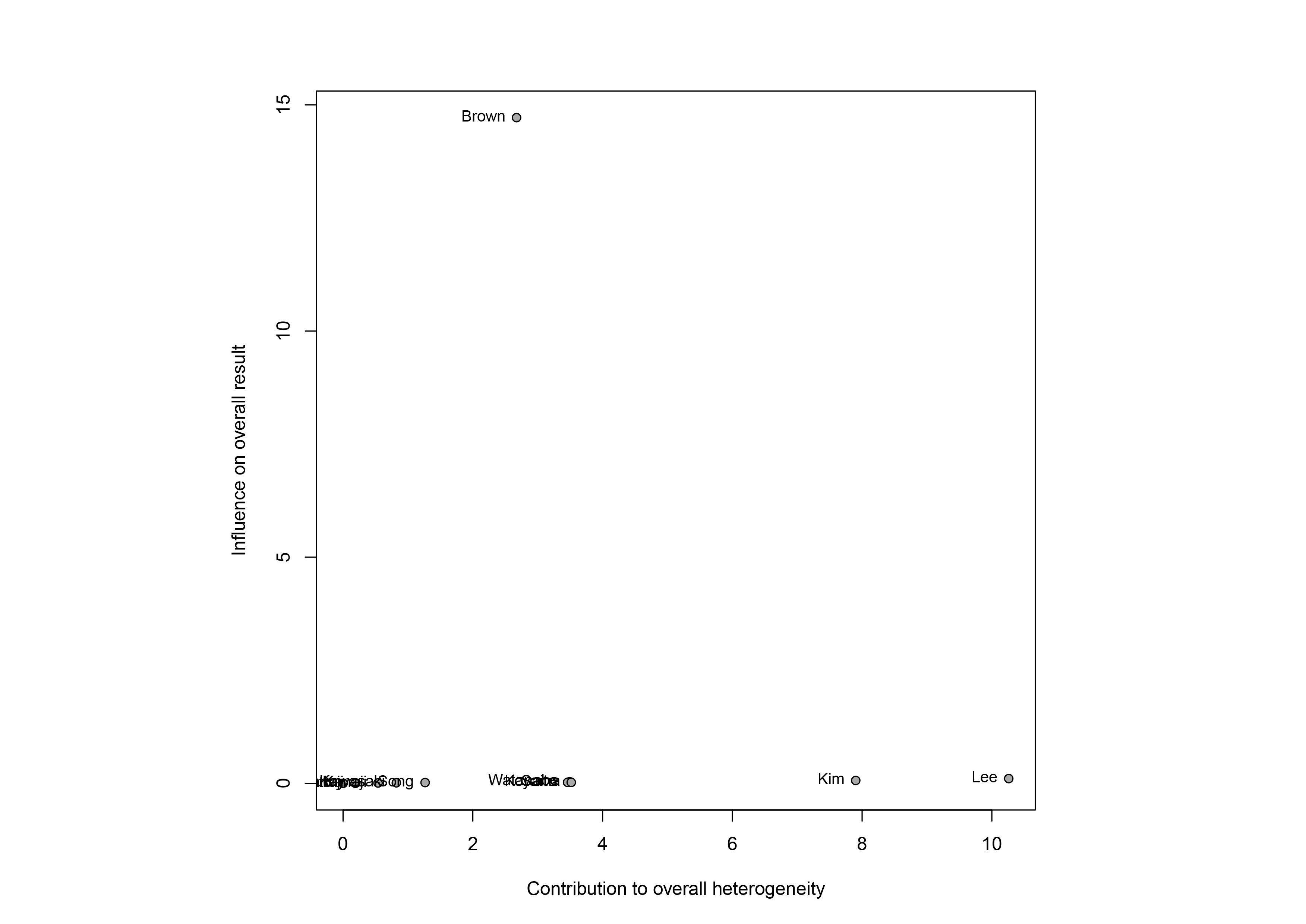

Supplement: S1 Fig — (TIF) [file pone.0259784.s001.tif]

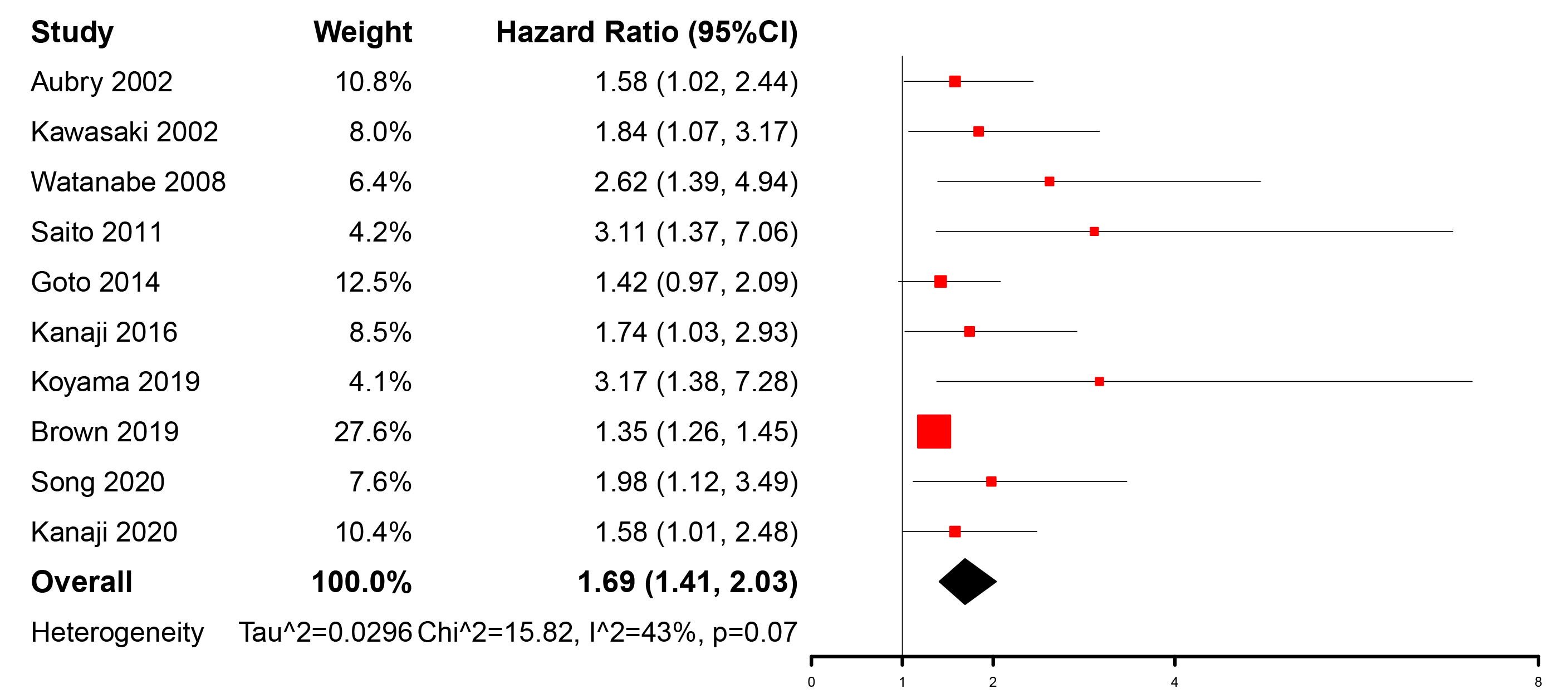

Supplement: S2 Fig — (TIF) [file pone.0259784.s002.tif]

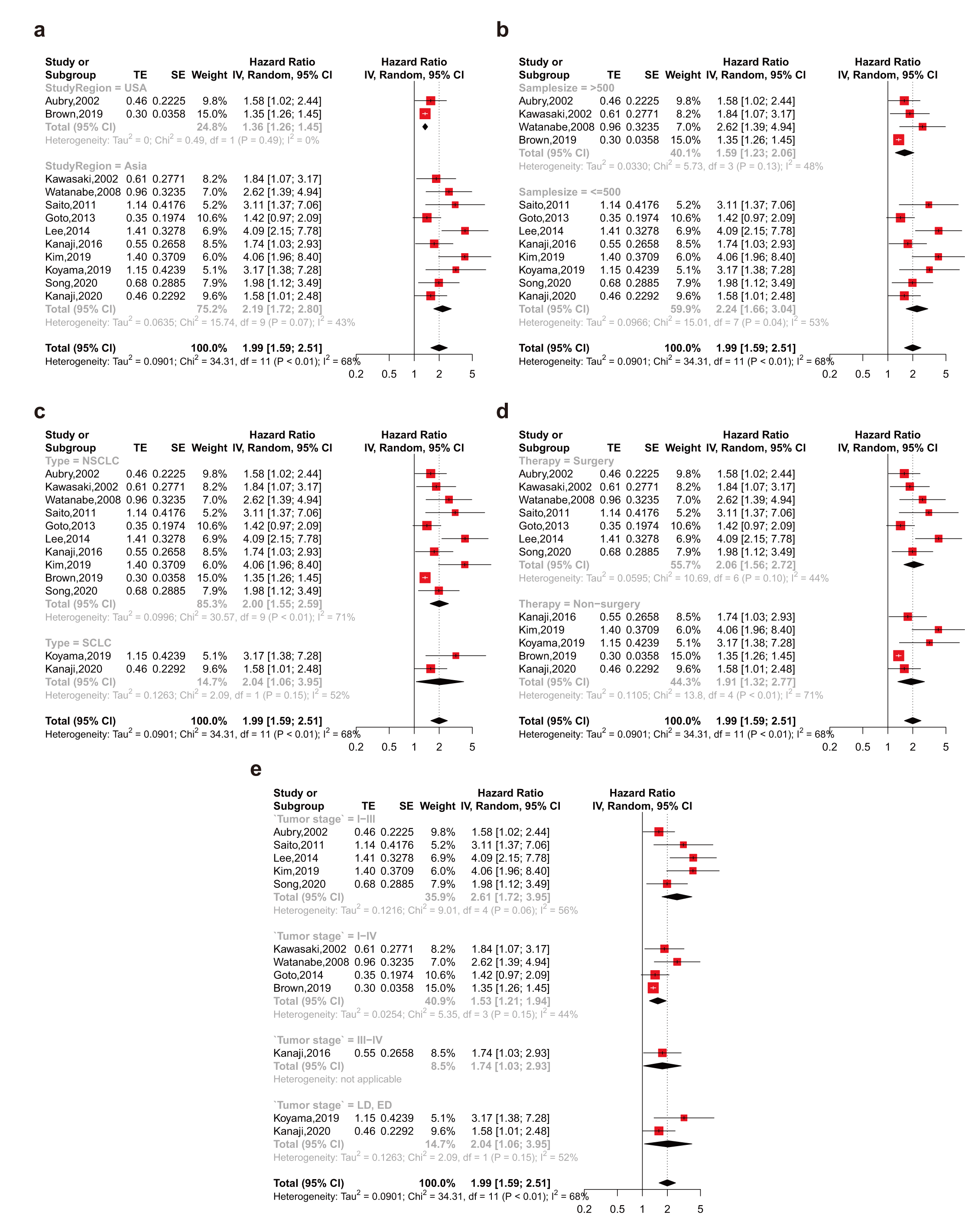

Supplement: S3 Fig — The subgroup analyses stratified by (a) study region, (b) sample size, (c) tumor histology, (d) therapy, and (e) tumor stage. (TIF) [file pone.0259784.s003.tif]
