# Supplementary material for: Impact of concomitant idiopathic pulmonary fibrosis on prognosis in lung cancer patients: A meta-analysis
Source: PLoS One. 2021 Nov 12;16(11):e0259784. doi: 10.1371/journal.pone.0259784 (PMC8589161; doi:10.1371/journal.pone.0259784)
Supplement: S1 Table — (DOCX) [file pone.0259784.s004.docx]

**S6 File**

**the results of the quality assessment for each individual study**

|  | Selection | | | | Comparability | | Exposure | | |  |  |
| --- | --- | --- | --- | --- | --- | --- | --- | --- | --- | --- | --- |
| Assessment criteria | Case definition adequate | Representativeness  of the cases | Selection of controls | Definition of controls | Comparability based on design or analysis | | Ascertainment of exposure | Same method of ascertainment for cases and controls | Non-response rate | Outcome | Total |
| Aubry2002 | ● | ○ | ● | ● | ● | ● | ● | ● | ○ | OS | 7 |
| Kawasaki2002 | ● | ● | ● | ● | ● | ○ | ● | ● | ○ | OS, DFS | 7 |
| Watanabe2008 | ● | ○ | ● | ● | ● | ● | ● | ● | ○ | OS, DFS | 7 |
| Saito2011 | ● | ○ | ● | ● | ● | ● | ● | ● | ● | OS | 8 |
| Goto2014 | ● | ● | ● | ● | ● | ○ | ● | ● | ● | OS | 8 |
| Lee2014 | ● | ● | ● | ● | ● | ● | ● | ● | ○ | OS, RFS | 8 |
| Kanaji2016 | ● | ● | ● | ● | ● | ● | ○ | ● | ○ | OS, PFS | 7 |
| Kim2019 | ● | ○ | ● | ● | ● | ○ | ● | ● | ● | OS | 7 |
| Koyama2019 | ● | ● | ● | ● | ● | ● | ● | ● | ○ | OS, TTF | 8 |
| Brown2019 | ● | ● | ● | ● | ● | ● | ● | ● | ○ | OS, CSS | 8 |
| Song2020 | ● | ● | ● | ● | ● | ● | ● | ● | ● | OS, RFS | 9 |
| Kanaji2020 | ● | ● | ● | ● | ● | ○ | ● | ● | ○ | OS, PFS | 7 |

OS: overall survival; DFS: disease-free survival; RFS: recurrence-free survival; TTF: time to failure; CSS: cancer specific survival; PFS: progression-free survival
