# Supplementary material for: Impact of concomitant idiopathic pulmonary fibrosis on prognosis in lung cancer patients: A meta-analysis
Source: PLoS One. 2021 Nov 12;16(11):e0259784. doi: 10.1371/journal.pone.0259784 (PMC8589161; doi:10.1371/journal.pone.0259784)
Supplement: S2 File — (DOCX) [file pone.0259784.s006.docx]

**S2 File**

**Search strategy for meta-analysis of Impact of concomitant idiopathic pulmonary fibrosis on prognosis in lung cancer patients (PubMed via NLM)**

#1 Search (idiopathic pulmonary fibrosis[Title/Abstract]) OR (IPF[Title/Abstract])

#2 Search (lung cancer[Title/Abstract]) OR (pulmonary tumor[Title/Abstract]) OR (lung carcinoma[Title/Abstract])

#3 Search (prognosis[Title/Abstract]) OR (survival[Title/Abstract])

#4 Search #1 AND #2 AND #3
